# Diagnosis of endocarditis caused by *Mycobacterium* abscessus

**DOI:** 10.4103/0256-4947.67086

**Published:** 2010

**Authors:** Khalifa Al-Benwan, Suhail Ahmad, Eiman Mokaddas, Molly Johny, Madan M. Kapoor

**Affiliations:** a From the Department of Microbiology, Al-Amiri Hospital, Ministry of Health, Kuwait; b From the Department of Microbiology, Faculty of Medicine, Kuwait University, Kuwait; c From the Department of Medicine, Al-Amiri Hospital, Ministry of Health, Kuwait

## Abstract

We report a fatal case of native valve endocarditis due to *Mycobacterium abscessus* in a hemodialysis patient. The diagnosis was based on culture isolation of acid-fast bacilli from peripheral blood and a permanent catheter tip, and their identification as *M abscessus* by a reverse hybridization-based assay and direct DNA sequencing of the 16S-23S internal transcribed spacer region. Rapid diagnosis and combination therapy are essential to minimize mortality due to this pathogen. Although combination therapy was started with clarithromycin and tigecycline, the patient refused to take clarithromycin due to severe abdominal pain. The patient became afebrile after therapy with tigecycline alone although bacteremia persisted. He was discharged against medical advice and readmitted three months later for persistent fever. His blood cultures again yielded *M abscessus* and a transesophageal echocardiogram showed two mobile vegetations. The patient was noncompliant with therapy and died due to cardiac arrest and multiorgan failure. This report shows that *M abscessus* should also be considered in the differential diagnosis of infective endocarditis in hemodialysis patients.

Clinical syndromes caused by nontuberculous mycobacteria (NTM) have gained attention since the 1980s due to the increase in disseminated infections in association with the human immunodeficiency virus (HIV) epidemic.^1^,^2^ Nontuberculous mycobacterial disease occurs infrequently in immunocompetent individuals.^2^ Despite their ubiquitous presence in soil and water, the occurrence of various NTM species varies greatly by geographic region. Based on their growth rates, NTM have been classified into slowly growing or rapidly growing mycobacteria. Rapidly growing mycobacteria grow within seven days on solid media.^3^ More than 70% of rapidly growing mycobacterial infections are attributed to *Mycobacterium abscessus, Mycobacterium fortuitum, and Mycobacterium chelonae*.^4^ Species-specific identification is critical for treatment selection and prognosis; however, conventional methods are time-consuming and do not reliably differentiate some species such as *M abscessus* and *M chelonae*.^3^ We report a rare case of native valve endocarditis in a dialysis patient with no previous history of abnormal valves or heart murmur. Application of molecular methods identified the etiological agent as *M abscessus*.

## CASE

A 54-year-old Kuwaiti man was admitted to Al-Amiri (a major tertiary care) hospital in May 2007 with a 4-week history of progressive general weakness, fever, and weight loss. The underlying conditions included chronic liver disease secondary to chronic hepatitis C and alcoholic cirrhosis, end-stage renal disease requiring hemodialysis for two years, and chronic obstructive pulmonary disease. His vital signs were as follows: temperature 38.1°C, pulse 90/min, blood pressure 140/70 mm Hg and respiratory rate 20/min. Also noted were an ejection systolic murmur (grade 2/6) over the aortic area and a pansystolic murmur at the apex radiating to the axilla. There were no skin lesions or other abnormalities.

Laboratory investigations revealed a peripheral WBC count of 4.7×10 ^9^ /L (83% polymorphs), hemoglobin at 9.1 g/dL, erythrocyte sedimentation rate of 80 mm/h, and a platelet count of 107×10 ^9^ /L. Chest radiography and urine analysis results were normal, and tests for antibodies to hepatitis B and HIV, a skin test for tuberculosis and tumor markers for abdominal malignancy were negative. Urea (20.4 mmol/L) and creatinine (721 μmol/L) levels were elevated; electrolyte and cardiac enzyme levels and liver function tests were normal. Blood was drawn on five separate days spanning 9 days through vein puncture (peripheral blood), perm-catheter and peripheral catheter. Cultures of these blood samples and culture of the perm-catheter tip yielded gram-positive beaded bacilli within 60-110 hours in a BACTEC 9240 system (Becton Dickenson). Two types of colonies (smooth and rough morphology) grew on chocolate agar, both showing gram-positive beaded bacilli. Ziehl-Neelsen stain of all cultured organisms showed acid-fast bacilli, suggesting mycobacterial infection.

**Figure 1:**
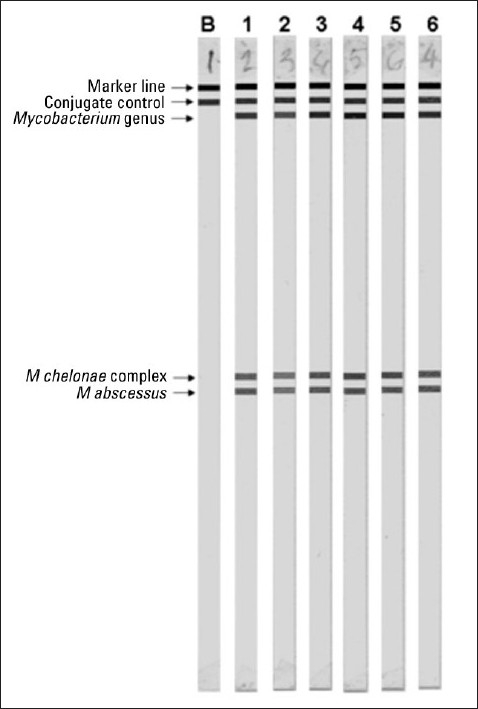
The INNO-LiPA mycobacteria v2 hybridization patterns for control of kit components (lane B) and for *M abscessus* isolates grown from blood samples obtained through peripheral catheter (lanes 1 and 2), venous puncture (lane 3), perm-catheter (lane 4), from perm-catheter tip (lane 5) and from blood sample obtained through peripheral catheter during second hospitalization (lane 6). The positions of marker line for alignment, conjugate control for the test of kit components and probes for detection of *Mycobacterium* genus-specific, *M chelonae* complex-specific, and *M abscessus*-specific DNA included on the strips are marked.

DNA from the isolates was prepared by the boiling method and treated with Chelex-100 to remove PCR inhibitors, as described in detail previously.^5^ Specific amplification of an rpoB-derived 136 bp fragment in a multiplex PCR carried out as described previously,^6^ identified the isolates as NTM. A commercial reverse hybridization-based (INNO-LiPA Mycobacteria v2, Innogenetics, Belgium) assay was used, as described in detail previously,^7^ which identified all the cultured isolates as being *M abscessus* (Figure 1). The results were confirmed by DNA sequencing of species-specific 16S-23S internal transcribed spacer (ITS) region for two blood culture isolates and for the isolate grown from the perm-catheter tip. The ITS region was amplified as described previously,^7^ except that panmycobacterial primers (MYCF, 5’-GATTGGGACGAAGTCGTAACAAG-3’ and MYCR, 5’-CTCGGTTGACAGCTCCCCGAG-3’) derived from 16S and 23S rRNA genes were used. The amplified fragments were purified and both strands were sequenced by using amplification primers and a cycle DNA sequencing kit (DTCS CEQ 2000, Beckman-Coulter) as described previously.^7^,^8^ The DNA sequences of the 16S-23S ITS region from all the three isolates were completely identical (EMBL accession nos. FM955484-FM955486) and showed 100% identity with the corresponding sequences from several *M abscessus* strains in BLAST (http://www.ncbi.nlm.nih.gov/BLAST/Blast.cgi) searches, which confirmed their identity as *M abscessus*. The organism was susceptible to amikacin, clarithromycin, imipenem, and tigecycline, but resistant to streptomycin, isoniazid, rifampicin, ethambutol, and pyrazinamide.

Following specific diagnosis, the perm-catheter was removed and a femoral catheter was fixed for dialysis. Treatment was started with intravenous clarithromycin (500 mg twice daily) and tigecycline (50 mg twice daily), but the patient refused to take clarithromycin after two days due to severe abdominal pain. Despite monodrug therapy with tigecycline for two weeks, the patient became afebrile and although bacteremia persisted and follow-up blood cultures again yielded *M abscessus*, the patient was discharged against medical advice. He was readmitted three months later and two sets of blood cultures again yielded *M abscessus* (Figure 1) with the same susceptibility profile as recorded earlier. Transthoracic echocardiography revealed aortic sclerosis, concentric left ventricular hypertrophy (LVH), and thick mitral annular calcification with a high suspicion of vegetation on the anterior mitral valve leaflet (AMVL). Transesophageal echocardiography showed a grossly distorted mitral valve (MV), moderate mitral regurgitation, and two mobile echodense vegetations of 0.6×0.6 cm on the atrial side of AMVL and the base of the aortic valve. Based on blood cultures and transesophageal echocardiography, a clinical diagnosis was made of native valve endocarditis due to *M abscessus*. Treatment with clarithromycin and tigecycline was restarted, but the patient was noncompliant. Three weeks later, transesophageal echocardiography again showed significant increase in the size of the vegetations attached to the AMVL. Abdominal sonography revealed an enlarged liver, moderate splenomegaly, and free fluid in the peritoneal cavity. The ECG showed loss of the R wave and a tall peaked T-wave. The patient died due to cardiac arrest five weeks after the second hospitalization as a result of multi-organ failure.

## DISCUSSION

*Mycobacterium abscessus* is widespread in the environment and has been documented as a source of nosocomial infection in immunocompromised patients. The organism is capable of colonizing the respiratory tract and causes pulmonary disease in certain high-risk groups such as white females older than 60 years of age and patients with cystic fibrosis or prior pulmonary mycobacterial infection, particularly tuberculosis.^4^,^9^ Although *M abscessus* infections can occur in both immunocompetent and immunocompromised hosts, immune status and underlying comorbidities contribute to the disease severity, and disseminated infections are typically seen in patients with impaired immunity.^4^,^10^ *Mycobacterium abscessus* has the ability to form biofilms and to grow even in distilled water. Due to its occurrence in municipal water supply systems as a result of its resistance to common disinfectants, *M abscessus* may also cause cutaneous infections that usually resolve themselves but may occasionally also require medical or surgical treatment.^4^,^10^ *Mycobacterium abscessus* and its close relatives have also been found in water used in reprocessing hemodialyzers and have been associated with bacteremia and disseminated disease in hemodialysis patients.^11^,^12^ In an outbreak involving 27 hemodialysis patients, the source of infection was traced to exposure to processed hemodialyzers that contained viable, rapidly growing mycobacteria; 25 out of 27 patients were infected with *M abscessus*.^12^ Although not investigated, it is probable that the source of infection in the present case also involved the use of a hemodialyzer as skin lesions were absent and pulmonary involvement was not apparent.

Rapid diagnosis and prolonged combination therapy are mandatory for proper treatment and to minimize mortality as *M abscessus* is inherently resistant to multiple antibiotics.^2^,^4^ Species-specific identification of *M abscessus* by conventional methods is time-consuming and may not be reliable and recently developed molecular methods have provided more accurate identification. The present case was also identified by the application of molecular methods.

*M abscessus* strains isolated from patients with pulmonary involvement invariably show in vitro resistance to antituberculous drugs. Although treatment of such patients with standard antituberculous drug therapy results in considerable clinical and radiological improvement, *M abscessus*-specific treatment is required for complete resolution of the infection.^13^ Typically, antibiotics with maximum in vitro activity against *M abscessus* isolates include amikacin, clarithromycin, tigecycline, and cefoxitin. To a lesser extent, linezolid and imipenem are also effective against nearly 50% of clinical *M abscessus* isolates. However, in vitro drug susceptibility patterns do not always correlate with clinical response and measurements of minimum inhibitory concentrations of antibiotics do not always predict their therapeutic effects.^4^ Successful management of patients with disseminated infections usually involves prolonged combination therapy (>6 months) with a macrolide (clarithromycin) or amikacin plus imipenem, cefoxitin, or tigecycline.^4^,^10^ Combination therapy with clarithromycin and tigecycline was also started in our case, but the patient refused to take clarithromycin due to severe abdominal pain. Although removal of catheters and treatment with tigecycline reduced the bacterial load, bacteremia persisted, most likely due to the shorter duration of monodrug therapy that was insufficient to completely resolve the deep-seated infection (heart valves). Di Pentima et al^14^ diagnosed a case of sepsis (and presumed endocarditis) due to *M abscessus* in a very low birth weight neonate by high performance liquid chromatography analysis of fatty acids and successfully managed it by combination therapy. Although amikacin and cefoxitin have exhibited intermediate in vitro activity against *M abscessus*, prolonged combination therapy with these two agents has been reported to completely resolve the infection.^14^ Although noncompliance with the therapy regimen most likely resulted in a fatal outcome for our patient, endocarditis caused by *M abscessus* generally has very poor prognosis despite combination antimicrobial therapy as only two of the ten documented cases described in the literature have survived after treatment.^15^ In conclusion, *M abscessus* should be considered in the differential diagnosis of endocarditis in hemodialysis patients, and molecular methods are useful for its rapid and specific identification.
